# Voxelwise degree centrality alterations and cognitive performance in severe unilateral M1-segment middle cerebral artery stenosis with ipsilateral watershed infarction

**DOI:** 10.3389/fnagi.2026.1969092

**Published:** 2026-10-07

**Authors:** Zhilin Huang, Xiaoxue Liang, Liyong Li, Yude Wei, Yi Zhang, Wenqian Wei, Xuanmei Quan, Kanglei Li, Kunlong Yao, Jian Tang, Yueling Zhang, Jianyi Huang, Jian Zhang

**Affiliations:** 1 Department of Neurology, The Second Affiliated Hospital of Guangxi Medical University, Nanning, China; 2 Department of Geriatrics, The Second Affiliated Hospital of Guangxi Medical University, Nanning, China; 3 Department of Neurology, The People's Hospital of Chongzuo, Chongzuo, China

**Keywords:** cognitive performance, degree centrality, middle cerebral artery stenosis, resting-state functional magnetic resonance imaging, watershed infarction

## Abstract

**Purpose:**

To characterize voxelwise degree centrality (DC) alterations and their exploratory clinical associations in severe unilateral M1-segment middle cerebral artery stenosis with ipsilateral watershed infarction.

**Methods:**

This cross-sectional study included 40 patients with left-sided disease, 47 with right-sided disease, and 44 healthy controls. Resting-state functional magnetic resonance imaging-derived weighted DC (*r* > 0.25) was compared using analysis of covariance with adjustment for educational attainment, Tukey–Kramer testing, and Gaussian random field-corrected patient–control contrasts (two-tailed voxel *P* < 0.001; cluster *P* < 0.05). Exploratory clinical associations were assessed using Spearman rank correlation.

**Results:**

Mini-Mental State Examination (MMSE) scores differed among groups (*P* < 0.001), with both patient groups scoring lower than controls; Montreal Cognitive Assessment (MoCA) scores also differed (*P* < 0.001), with the left-sided group lowest, the right-sided group intermediate, and controls highest. The left-sided group showed lower DC predominantly in left temporal–opercular and postcentral–inferior parietal regions and higher DC in right medial/subcortical and posterior-midline regions; the largest lower-DC cluster peaked at MNI coordinates −48, −18, 39 (*Z* = −5.5420). The right-sided group showed lower DC in right putaminal/insular, temporal, and parietal–sensorimotor regions and higher DC in the left cerebellum; the largest lower-DC cluster peaked at MNI coordinates 48, −27, 36 (*Z* = −6.0021). In the right-sided group, exploratory nominal associations were observed between MMSE scores and DC in the left cerebellar, right superior temporal-pole, and right parietal–sensorimotor clusters (*r* = −0.300 to 0.421; nominal *P* = 0.003–0.040). No nominal cognitive–DC correlations were observed in the left-sided group, and no MoCA–DC correlations in the right-sided group.

**Conclusion:**

Distributed DC alterations accompanied cognitive performance differences, with selective exploratory nominal MMSE associations in right-sided disease. DC may merit further evaluation as a candidate imaging feature of BOLD-based functional alterations associated with cognitive differences.

## 1 Introduction

Severe unilateral stenosis of the M1 segment of the middle cerebral artery (MCA) accompanied by an ipsilateral watershed infarction represents a clinically relevant cerebrovascular phenotype. Although conventional neurological deficits may be relatively mild, cognitive impairment can remain clinically important because it may affect functional independence, treatment adherence, and participation in rehabilitation (El Husseini et al., 2023). Severe symptomatic intracranial atherosclerotic stenosis is also associated with an increased risk of recurrent ischemic events (Gutierrez et al., 2022; Turan et al., 2022). A more complete characterization of the functional consequences of this condition is therefore warranted.

Watershed infarcts involve cortical or internal border-zone regions at the distal margins of arterial territories (Dogariu et al., 2023). In patients with severe MCA stenosis, the location of the visible infarct and the angiographic severity of stenosis may not fully explain variation in cognitive performance (Kolskår et al., 2022; Ruan et al., 2026). Focal ischemic injury may also affect interconnected regions beyond the structural lesion, leading to distributed alterations in brain functional organization (Sagues et al., 2025). Although structural and vascular imaging provide essential information on lesion location and arterial disease, they offer limited insight into the contribution of individual regions to whole-brain functional networks. A network-based approach may therefore provide complementary information beyond lesion anatomy alone.

Resting-state functional magnetic resonance imaging (rs-fMRI) enables the investigation of spontaneous brain functional organization without requiring task performance. Degree centrality (DC) is a voxelwise, data-driven measure that quantifies the overall functional connectivity of each voxel with the rest of the brain. Weighted DC reflects the summed strength of suprathreshold correlations and can identify regions with relatively high or low whole-brain connectivity without requiring a predefined seed (Zuo et al., 2012). Previous resting-state studies of ischemic stroke have demonstrated distributed alterations in functional connectivity and centrality rather than abnormalities confined to the structural lesion. Altered DC has been reported after ischemic stroke and may be related to cognitive performance (Min et al., 2023), while acute stroke studies have demonstrated lesion-side-dependent and remote functional-connectivity alterations (Katsurayama et al., 2024). A recent systematic review further showed that post-stroke cognitive impairment is associated with distributed resting-state connectivity abnormalities across multiple cortical and subcortical systems, with both reduced connectivity and increases in remote or contralesional regions being reported (Sagues et al., 2025). These BOLD-based functional measures may additionally be influenced by stroke-related hemodynamic delay and altered cerebrovascular reactivity (Siegel et al., 2016; Wang et al., 2024). Accordingly, focal ischemic injury may be accompanied by lower functional centrality in affected or functionally connected regions and altered centrality in remote regions; however, such changes cannot be attributed exclusively to neuronal reorganization. Whole-brain DC alterations in severe unilateral M1-segment MCA stenosis accompanied by watershed infarction remain insufficiently characterized, and their relationship with cognitive performance is unclear.

In this study, we used voxelwise weighted DC analysis to characterize whole-brain functional centrality in patients with severe left-sided M1-segment MCA stenosis (MCAS-L) or right-sided M1-segment MCA stenosis (MCAS-R), each accompanied by an acute ipsilateral watershed infarction. Each patient group was compared with healthy controls (HCs), and associations between clusterwise DC values and cognitive performance were examined. Anxiety and depressive symptom scores were additionally evaluated in secondary exploratory analyses. We hypothesized that both patient groups would show distributed DC alterations, including lower DC in ipsilesional association and sensorimotor regions and possible increases in remote regions. We further hypothesized that variation in DC within selected regions might be associated with cognitive performance.

## 2 Materials and methods

### 2.1 Study design and participants

We conducted a single-center cross-sectional observational study in the Department of Neurology, Second Affiliated Hospital of Guangxi Medical University. Participants were recruited consecutively from March 1, 2025, through February 28, 2026. The institutional ethics committee approved the study, and all participants or their legally authorized representatives provided written informed consent.

Patients were eligible when they had unilateral severe stenosis of the M1 segment of the MCA and an acute ipsilateral watershed infarction confirmed on diffusion-weighted MRI. Watershed infarction was defined as acute ischemia in the cortical or internal border zones of the ipsilateral MCA territory, including cortical, internal, or mixed patterns. We excluded large territorial infarctions extending beyond the border zones, isolated perforator infarcts, suspected embolic lesions, and isolated stenosis of M2 or more distal MCA branches. Two experienced neuroradiologists, blinded to the clinical and functional-network data, independently reviewed diffusion-weighted images and classified the infarct pattern as cortical, internal, or mixed. Disagreements were resolved by consensus.

Stenosis was assessed by computed tomography angiography (CTA), magnetic resonance angiography (MRA), and/or digital subtraction angiography (DSA) according to the Warfarin-Aspirin Symptomatic Intracranial Disease (WASID) method (Chimowitz et al., 1995; Samuels et al., 2000), with severe disease defined as 70%–99% luminal narrowing. When multiple vascular examinations were available, DSA served as the reference; otherwise CTA was prioritized over MRA for lesion location and severity. Patients could not have moderate or greater stenosis in another intracranial or extracranial artery. They had to be able to complete magnetic resonance imaging (MRI) and clinical testing, have no severe disturbance of consciousness or psychiatric or behavioral abnormality that would invalidate assessment, and be right-handed according to the Edinburgh Handedness Inventory. MRI was obtained within 7 days after infarct onset.

Healthy controls had no history of stroke, transient ischemic attack, or another major neurological disorder; no moderate or greater intracranial or extracranial stenosis; no structural brain lesion on routine MRI; and right-handedness. Exclusion criteria for all participants were a previous large cortical infarct or intracerebral hemorrhage, another definite neurological disorder, severe cardioembolic risk or systemic disease, MRI contraindication, excessive head motion, or preprocessing failure.

### 2.2 Clinical and neuropsychological assessment

Clinical assessment was completed during the same acute-phase evaluation. Neurological deficit was rated with National Institutes of Health Stroke Scale (NIHSS) and global disability with the modified Rankin Scale (mRS). Global cognition was examined using the Mini-Mental State Examination (MMSE) and Montreal Cognitive Assessment (MoCA). Anxiety and depressive symptoms were assessed with the Hamilton Anxiety Rating Scale (HAMA) and Hamilton Depression Rating Scale (HAMD), respectively. Vascular history and risk-factor variables were included in the baseline dataset.

### 2.3 MRI acquisition

MRI data were acquired on a Siemens MAGNETOM Vida 3.0 T scanner. Participants remained awake with their eyes closed, stayed still, and avoided deliberate cognitive activity. High-resolution structural images were acquired using a three-dimensional T1-weighted magnetization-prepared rapid acquisition gradient-echo (MPRAGE) sequence with the following parameters: repetition time (TR), 2000 ms; echo time (TE), 1.9 ms; inversion time (TI), 950 ms; flip angle, 8°; field of view (FOV), 256 × 256 mm; acquisition matrix, 256 × 256; voxel size, 1.0 × 1.0 × 1.0 mm^3^; slice thickness, 1.0 mm; 176 slices; and acquisition time, 3 min. Resting-state blood oxygen level-dependent (BOLD) images were acquired using a gradient-echo-planar imaging sequence with the following parameters: TR, 2000 ms; TE, 30 ms; flip angle, 90°; FOV, 224 × 224 mm; acquisition matrix, 64 × 64; voxel size, 3.5 × 3.5 × 3.5 mm^3^; slice thickness, 3.5 mm; 32 slices; 240 vol; and acquisition time, 8 min 6 s.

### 2.4 rs-fMRI preprocessing

Preprocessing of rs-fMRI data was performed using the advanced edition of the Data Processing Assistant for Resting-State fMRI (DPARSFA) module in Data Processing & Analysis for Brain Imaging (DPABI_V9.0_250415), running on MATLAB R2022a (MathWorks, Natick, MA, USA^1^) and based on Statistical Parametric Mapping 12 (SPM12; Wellcome Centre for Human Neuroimaging, UCL Queen Square Institute of Neurology, London, UK^2^) (Chao-Gan and Yu-Feng, 2010; Zhang et al., 2025a).

The first 10 vol were discarded. Slice-timing correction was applied to the 32-slice interleaved acquisition, followed by realignment for head-motion correction. Datasets with head motion exceeding 3.0 mm of translation or 3° of rotation were excluded. Structural and functional images were coregistered, and spatial normalization to Montreal Neurological Institute space was performed using diffeomorphic anatomical registration through exponentiated Lie algebra (DARTEL) implemented in DPABI. Normalized functional images were resampled to 3 × 3 × 3 mm^3^ isotropic voxels. White matter and cerebrospinal fluid signals and Friston-24 motion parameters were regressed out (Friston et al., 1996; Yan et al., 2013), with a constant and linear trend included in the nuisance-regression model. Global signal regression was not performed. Vascular and physiological effects on BOLD signals could not be fully removed (Siegel et al., 2016; Chen and Gauthier, 2021; Amemiya et al., 2022; Wang et al., 2024). The residual time series were temporally band-pass filtered at 0.01–0.1 Hz. Spatial smoothing was not applied to the functional time series before DC calculation. All preprocessed images underwent visual quality inspection. Mean framewise displacement (FD) was retained as a motion covariate.

### 2.5 Degree centrality calculation

Degree centrality is a graph theory metric quantifying the importance of a brain region within the global functional network, calculated based on voxel-wise functional connectivity strength (Zuo et al., 2012; Min et al., 2023). Voxel-wise functional connectivity matrix was constructed by calculating Pearson correlation coefficients between all possible pairs of voxels within the DPABI brain mask. For each voxel, weighted DC was defined as the sum of positive Pearson correlation coefficients exceeding the predefined correlation threshold of *r* > 0.25. Individual voxel-to-voxel Pearson correlation coefficients were not Fisher r-to-z transformed before weighted summation. Finally, individual degree centrality maps were converted to *Z* values to mitigate the influence of intersubject global signal variations. The resulting standardized DC maps were subsequently spatially smoothed using a 4-mm FWHM Gaussian kernel.

### 2.6 Statistical analysis

Baseline demographic and clinical characteristics were analyzed using Statistical Package for the Social Sciences (SPSS) version 26.0 (IBM Corp., Armonk, NY, USA). Continuous variables were compared using one-way analysis of variance or Kruskal-Wallis tests according to distribution, and categorical variables using chi-square or Fisher exact tests. Education was treated as a four-category variable. Pairwise comparisons used Bonferroni correction; two-sided *P* < 0.05 was considered significant.

For the voxelwise analysis of the resting-state fMRI data, standardized DC maps were compared among the three groups using one-way analysis of covariance (ANCOVA) implemented in the statistical analysis module of DPABI. Age, sex, previous stroke, hypertension, diabetes mellitus, hyperlipidemia, mean framewise displacement, and educational attainment were included as covariates. Educational attainment was recorded in four ordered categories (primary school, junior high school, high school, and university) and was entered as an ordinal covariate. Following the omnibus ANCOVA, all three pairwise group comparisons were generated using the Tukey–Kramer honestly significant difference procedure while retaining the same covariate structure. In accordance with the primary study objective, the subsequent voxelwise analyses focused on the two patient–control comparisons: MCAS-L versus HCs and MCAS-R versus HCs. The statistical map for each patient–control comparison was separately corrected for spatial multiple comparisons using Gaussian random-field (GRF) theory, with a two-tailed voxel-level threshold of *P* < 0.001 and a cluster-level threshold of *P* < 0.05. GRF correction was performed within the prespecified brain mask, with spatial smoothness estimated separately from each statistical image. The resulting minimum cluster extents were 50 voxels for MCAS-L versus HCs and 53 voxels for MCAS-R versus HCs. Anatomical labels were assigned using the Automated Anatomical Labeling (AAL) atlas (Tzourio-Mazoyer et al., 2002), and peak Montreal Neurological Institute (MNI) coordinates, cluster extents, directions of effect, and peak *Z* values were obtained from the GRF-corrected cluster reports.

Significant clusters were identified from the GRF-corrected cluster reports, and the mean DC values within these clusters were extracted for correlation analyses. Within each patient subgroup, associations between the extracted DC values and clinical measures, including neurological deficit and functional disability (NIHSS and mRS), cognitive performance (MMSE and MoCA), and anxiety and depressive symptoms (HAMA and HAMD), were assessed using exploratory Spearman rank correlation analysis. Spearman rank correlation coefficients are reported as r. All correlations were two-tailed. Because these clusterwise clinical analyses were secondary and exploratory, exact nominal *P* values are reported, with nominal *P* < 0.05 used to identify exploratory associations; no multiplicity-adjusted threshold was used for confirmatory inference. To assess robustness to the correlation threshold used for weighted DC, the voxelwise analyses were repeated using *r* > 0.20 and *r* > 0.30, while all other preprocessing, covariate, group-comparison, and GRF-correction procedures were kept unchanged. To provide descriptive functional-network context, the peak coordinates of the final GRF-corrected clusters were additionally mapped onto the Yeo 2011 seven-network cortical parcellation (Yeo et al., 2011); peaks outside the cortical parcellation were recorded as not assigned.

## 3 Results

### 3.1 Participant characteristics

The final analysis included 131 participants: 40 patients with left-sided severe M1-segment MCA stenosis and ipsilateral watershed infarction (MCAS-L), 47 with right-sided disease (MCAS-R), and 44 healthy controls (HCs). Age, sex, education, diabetes mellitus, and hyperlipidemia did not differ significantly among the three groups (all *P* > 0.05; Table 1). Significant overall group differences were observed for previous stroke and hypertension (*P* < 0.001 and *P* = 0.001, respectively). In post hoc comparisons, both patient groups had a higher prevalence of previous stroke and hypertension than HCs, whereas MCAS-L and MCAS-R did not differ significantly. NIHSS and mRS scores also differed significantly among the groups (both *P* < 0.001; Table 1). Both patient groups had higher scores than HCs, whereas MCAS-L and MCAS-R did not differ significantly in post hoc comparisons.

**Table 1:** Demographic, vascular risk, clinical, and neuropsychological characteristics.

Variable	MCAS-L (*n* = 40)	MCAS-R (*n* = 47)	HC (*n* = 44)	*P* value	Post hoc comparisons
Age, mean ± SD, years	60.3 ± 8.4	58.9 ± 9.8	59.5 ± 8.6	0.783	
Sex, male, *n* (%)	28 (70.0)	38 (80.9)	33 (75.0)	0.499	
History of stroke, yes, *n* (%)	19 (47.5)	14 (29.8)	0 (0.0)	<0.001	a, b
Hypertension, yes, *n* (%)	30 (75.0)	32 (68.1)	17 (38.6)	0.001	a, b
Diabetes mellitus, yes, *n* (%)	11 (27.5)	9 (19.1)	8 (18.2)	0.522	
Hyperlipidemia, yes, *n* (%)	14 (35.0)	9 (19.1)	17 (38.6)	0.100	
Education level distribution, *n* (%)				0.191	
Education, primary school, *n* (%)	17 (42.5)	12 (25.5)	10 (22.7)		
Education, junior high school, *n* (%)	11 (27.5)	15 (31.9)	17 (38.6)		
Education, high school, *n* (%)	6 (15.0)	15 (31.9)	8 (18.2)		
Education, university, n (%)	6 (15.0)	5 (10.6)	9 (20.5)		
mRS, median (IQR)	1.0 (1.0)	1.0 (0.0)	0 (0.0)	<0.001	a, b
NIHSS, median (IQR)	0.0 (2.0)	0.0 (1.0)	0 (0.0)	<0.001	a, b
MMSE, median (IQR)	24 (8.0)	26 (4.0)	28.5 (2.0)	<0.001	a, b
MoCA, mean ± SD	16.0 ± 7.0	20.0 ± 4.4	23.0 ± 3.6	<0.001	a, b, c
HAMA, median (IQR)	3.0 (8.0)	3 (3.0)	2.5 (6.0)	0.426	
HAMD, median (IQR)	2.0 (5.0)	3 (4.0)	2 (5.0)	0.830	

Data are mean ± SD, median (interquartile range [IQR]), or *n* (%). Continuous variables were compared using one-way analysis of variance or Kruskal-Wallis tests; categorical variables using chi-square or Fisher exact tests. Education was analyzed as a single four-category variable; category-specific *P* values are not shown. Pairwise comparisons used Bonferroni correction. a, MCAS-L vs HC; b, MCAS-R vs HC; c, MCAS-L vs MCAS-R. *P* < 0.05 was significant. Variables with overall *P* < 0.05 but no significant pairwise differences after Bonferroni correction are left unmarked. MCAS-L, left-sided middle cerebral artery stenosis group; MCAS-R, right-sided middle cerebral artery stenosis group; HC, healthy control; mRS, modified Rankin Scale; NIHSS, National Institutes of Health Stroke Scale; MMSE, Mini-Mental State Examination; MoCA, Montreal Cognitive Assessment; HAMA, Hamilton Anxiety Rating Scale; HAMD, Hamilton Depression Rating Scale.

### 3.2 Cognitive and emotional assessments

Cognitive performance differed significantly among the three groups (Table 1). Median MMSE scores were 24 (interquartile range [IQR], 8.0) in MCAS-L, 26 (IQR, 4.0) in MCAS-R, and 28.5 (IQR, 2.0) in HCs (*P* < 0.001). Both patient groups had lower MMSE scores than HCs, whereas MCAS-L and MCAS-R did not differ significantly in post hoc comparisons. Mean MoCA scores were 16.0 ± 7.0, 20.0 ± 4.4, and 23.0 ± 3.6, respectively (*P* < 0.001). All three Bonferroni-adjusted pairwise comparisons were significant, with lower MoCA scores in MCAS-L than in MCAS-R and HCs and lower scores in MCAS-R than in HCs. In contrast, HAMA and HAMD scores did not differ significantly among the three groups (*P* = 0.426 and *P* = 0.830, respectively; Table 1).

### 3.3 Degree centrality differences

At the GRF-corrected threshold of two-tailed voxel-level *P* < 0.001 and cluster-level *P* < 0.05, the MCAS-L versus HC comparison identified five significant clusters (Table 2; Figure 1). Lower DC was observed predominantly in the left Rolandic operculum, middle temporal, and postcentral/inferior parietal regions, whereas higher DC was observed in right medial/subcortical and posterior-midline regions. The largest lower-DC cluster comprised 424 voxels and peaked at MNI coordinates −48, −18, 39 (*Z* = −5.5420).

**Figure 1:**
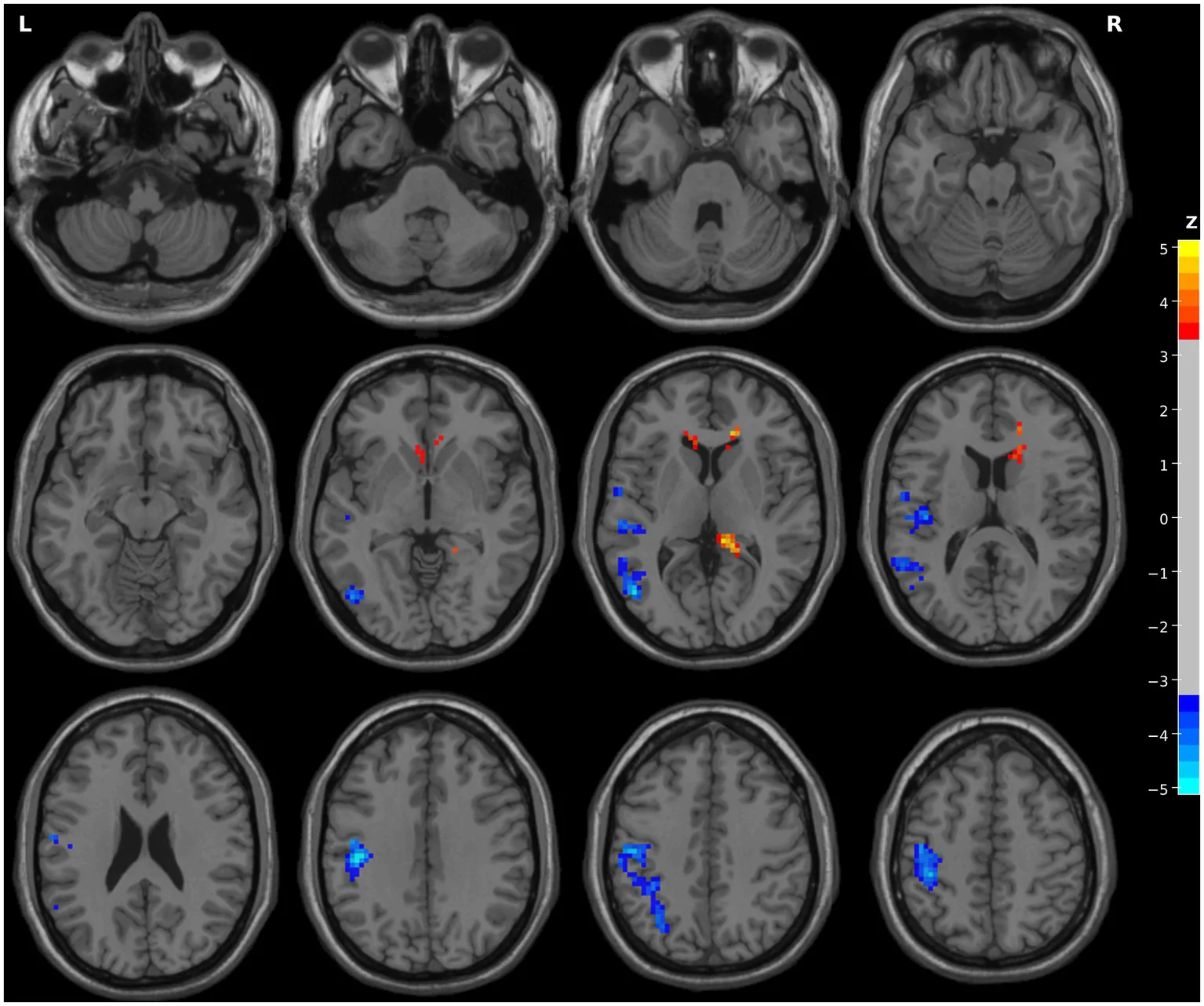
Degree centrality differences between the left-sided middle cerebral artery stenosis group and healthy controls. Axial maps show clusters with significantly higher degree centrality (warm colors) or lower degree centrality (cool colors) in the left-sided group relative to healthy controls after Gaussian random field correction (two-tailed voxel-level *P* < 0.001; cluster-level *P* < 0.05). Cluster details are provided in Table 2. The color bar represents *Z* values; L, left; R, right.

**Table 2:** Degree centrality differences in the GRF-corrected patient-control contrasts.

Contrast	Cluster	Direction	Principal AAL label	Hemisphere	Cluster extent, voxels	Peak MNI (*x*, *y*, *z*)	Peak *Z*
MCAS-L vs. HC	1	Increased	No AAL label at peak	Right	94	(15, 33, 9)	5.0290
MCAS-L vs. HC	2	Increased	No AAL label at peak	Right	54	(9, −36, 6)	4.9830
MCAS-L vs. HC	3	Decreased	Rolandic_Oper_L	Left	67	(−42, −21, 15)	−4.6469
MCAS-L vs. HC	4	Decreased	Temporal_Mid_L	Left	177	(−48, −69, 6)	−5.2476
MCAS-L vs. HC	5	Decreased	Postcentral_L	Left	424	(−48, −18, 39)	−5.5420
MCAS-R vs. HC	1	Increased	Cerebelum_8_L	Left	241	(−6, −72, −42)	5.1135
MCAS-R vs. HC	2	Decreased	No AAL label at peak	Right	114	(33, −6, −12)	−5.6122
MCAS-R vs. HC	3	Decreased	Temporal_Pole_Sup_R	Right	109	(60, 3, −6)	−5.2127
MCAS-R vs. HC	4	Decreased	Temporal_Mid_R	Right	160	(54, −54, 9)	−5.2924
MCAS-R vs. HC	5	Decreased	Temporal_Sup_R	Right	180	(54, −33, 12)	−5.5670
MCAS-R vs. HC	6	Decreased	SupraMarginal_R	Right	400	(48, −27, 36)	−6.0021

Peak anatomical labels were assigned according to the AAL atlas when the peak MNI coordinate fell within an AAL parcel. Peaks not assigned to an AAL parcel are reported as “No AAL label at peak.” Cluster extent represents the total number of voxels within each GRF-corrected cluster. Positive and negative peak *Z* values indicate higher and lower DC, respectively, in the patient group relative to healthy controls. Each patient–control contrast was corrected separately using GRF theory at a two-tailed voxel-level threshold of *P* < 0.001 and cluster-level *P* < 0.05. AAL, Automated Anatomical Labeling; DC, degree centrality; GRF, Gaussian random field; HC, healthy controls; MCAS-L, left-sided middle cerebral artery stenosis group; MCAS-R, right-sided middle cerebral artery stenosis group; MNI, Montreal Neurological Institute.

The MCAS-R versus HC comparison identified six significant clusters (Table 2; Figure 2). Lower DC was observed predominantly in the right putaminal/insular, superior and middle temporal, and supramarginal/parietal–sensorimotor regions, whereas higher DC was observed in the left cerebellum. The largest lower-DC cluster comprised 400 voxels and peaked at MNI coordinates 48, −27, 36 (*Z* = −6.0021). The higher-DC cerebellar cluster comprised 241 voxels and peaked at MNI coordinates −6, −72, −42 (*Z* = 5.1135).

**Figure 2:**
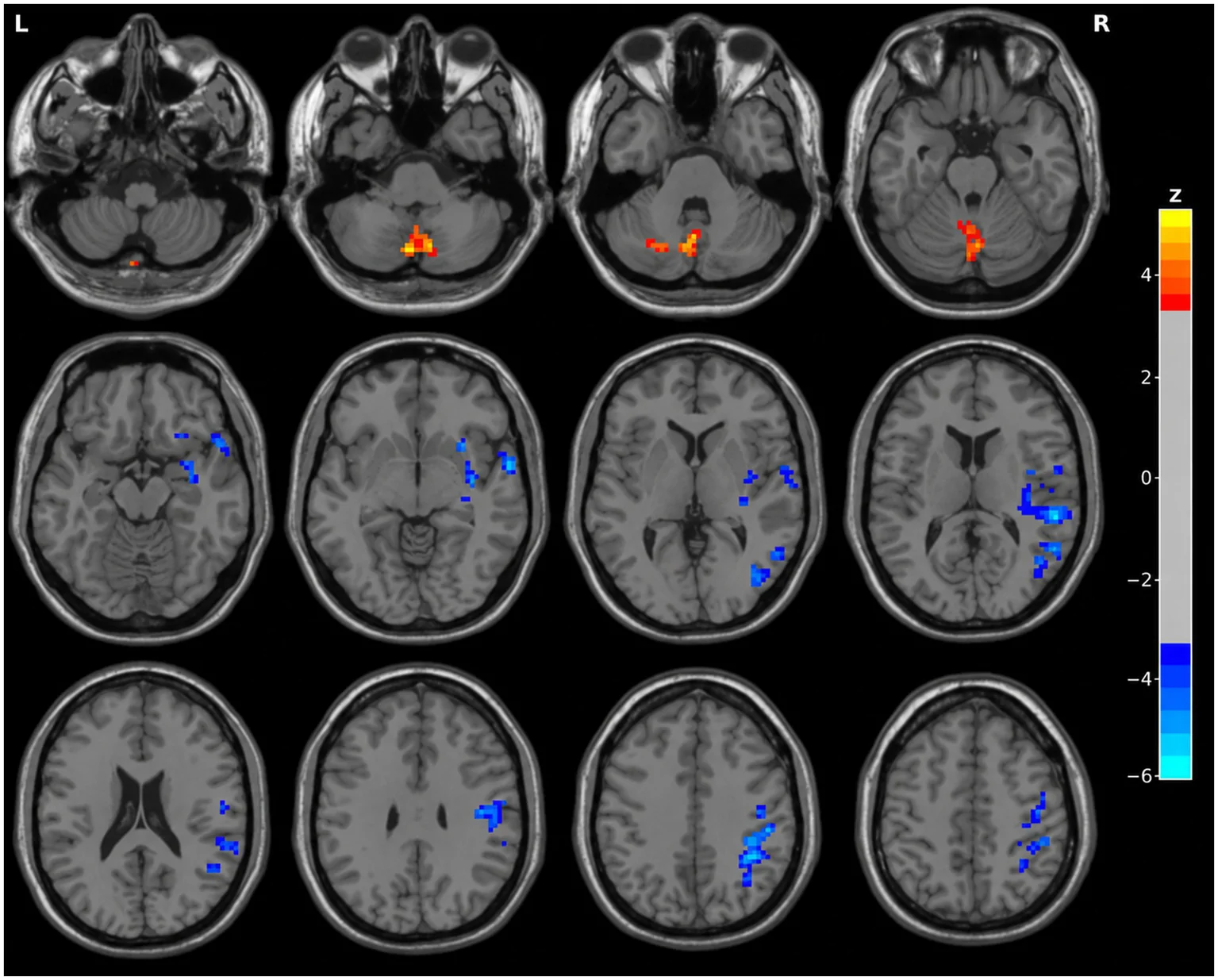
Degree centrality differences between the right-sided middle cerebral artery stenosis group and healthy controls. Axial maps show clusters with significantly higher degree centrality (warm colors) or lower degree centrality (cool colors) in the right-sided group relative to healthy controls after Gaussian random field correction (two-tailed voxel-level *P* < 0.001; cluster-level *P* < 0.05). Cluster details are provided in Table 2. The color bar represents *Z* values; L, left; R, right.

The principal lateralized spatial patterns were broadly preserved in sensitivity analyses using alternative weighted-DC correlation thresholds of *r* > 0.20 and *r* > 0.30, although some secondary cluster characteristics varied across thresholds (Supplementary Table S3). Detailed anatomical composition of the final clusters is provided in Supplementary Table S1, and descriptive Yeo-7 network affiliations are provided in Supplementary Table S2.

### 3.4 Exploratory associations with clinical assessments

Exploratory two-sided Spearman correlations between clusterwise mean DC values and clinical assessments are summarized in Table 3. No nominal clusterwise clinical associations were observed in MCAS-L. In MCAS-R, MMSE scores were inversely associated with the higher-DC left cerebellar cluster (cluster 1; *r* = −0.300, nominal *P* = 0.040) and positively associated with the lower-DC right superior temporal-pole cluster (cluster 3; *r* = 0.334, nominal *P* = 0.022) and the right parietal–sensorimotor/supramarginal cluster (cluster 6; *r* = 0.421, nominal *P* = 0.003) (Figure 3). HAMD scores showed a positive nominal association with the right middle temporal cluster (cluster 4; *r* = 0.297, nominal *P* = 0.042). No nominal MoCA–DC associations were observed in either patient subgroup. These associations are presented as secondary exploratory findings.

**Figure 3:**
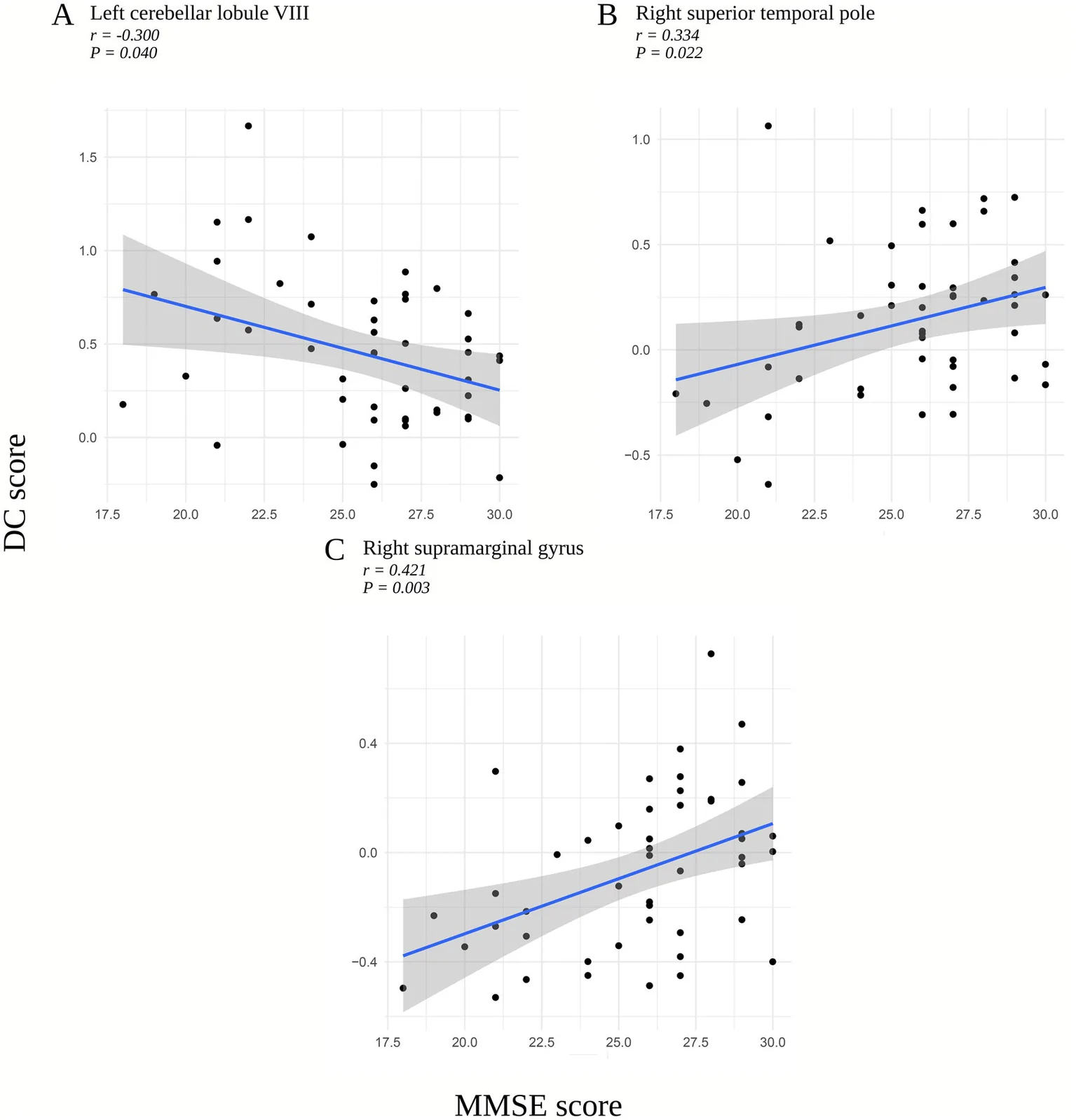
Exploratory associations between Mini-Mental State Examination (MMSE) scores and clusterwise mean degree centrality (DC) in the right-sided middle cerebral artery stenosis group (*n* = 47). **(A)** Left cerebellar cluster (cluster 1); **(B)** right superior temporal-pole cluster (cluster 3); and **(C)** right parietal–sensorimotor/supramarginal cluster (cluster 6). Associations were assessed using two-sided Spearman rank correlations, and exact nominal *P* values are shown. Fitted lines and shaded confidence bands are displayed for visualization. *r*, Spearman rank correlation coefficient.

**Table 3:** Exploratory correlations between clusterwise DC and clinical measures.

Cohort	Test	Scale	Cluster (direction)	Peak AAL label	Peak MNI	Coefficient	*P* value
MCAS-R	Spearman *r*	MMSE	1 (increased)	Cerebelum_8_L	(−6, −72, −42)	*r* = −0.300	0.040
MCAS-R	Spearman *r*	MMSE	3 (decreased)	Temporal_Pole_Sup_R	(60, 3, −6)	*r* = 0.334	0.022
MCAS-R	Spearman *r*	MMSE	6 (decreased)	SupraMarginal_R	(48, −27, 36)	*r* = 0.421	0.003
MCAS-R	Spearman *r*	HAMD	4 (decreased)	Temporal_Mid_R	(54, −54, 9)	*r* = 0.297	0.042

Only exploratory associations with nominal two-sided *P* < 0.05 are shown. Correlations were assessed using Spearman rank correlation separately within MCAS-L and MCAS-R. Exact nominal *P* values are reported; no multiplicity-adjusted threshold was used for confirmatory inference.

## 4 Discussion

### 4.1 Principal findings

In this study of severe unilateral M1-segment MCA stenosis with ipsilateral watershed infarction, several findings were prominent. First, cognitive performance differed substantially among the groups despite relatively mild neurological deficits. Both patient groups had lower MMSE scores than HCs, whereas MoCA showed a graded pattern, with the lowest scores in MCAS-L, intermediate scores in MCAS-R, and the highest scores in HCs. In contrast, HAMA and HAMD scores did not differ significantly among the groups. Second, both patient–control contrasts showed distributed and lateralized differences in voxelwise DC. MCAS-L was characterized predominantly by lower DC in left temporal–opercular and postcentral–inferior parietal regions, together with higher DC in right medial/subcortical and posterior-midline regions. MCAS-R showed lower DC in right putaminal/insular, temporal, and parietal–sensorimotor regions, whereas higher DC was observed in the left cerebellum. The principal spatial patterns were broadly preserved across alternative DC correlation thresholds. Third, clusterwise clinical associations were selective and exploratory. No nominal cognitive–DC associations were observed in MCAS-L, whereas MMSE performance in MCAS-R showed nominal associations with selected cerebellar, temporal, and parietal–sensorimotor clusters. A single nominal HAMD–DC association was also observed in MCAS-R.

### 4.2 Distributed DC alterations

DC characterizes the functional centrality of individual voxels according to the strength of their connections with the rest of the brain (Zuo et al., 2012). Accordingly, reduced DC indicates lower BOLD-based functional centrality relative to other voxels within the analysis mask and should not be interpreted as direct evidence of local neuronal inactivity. In MCAS-L, lower DC in the left temporal–opercular and postcentral–inferior parietal regions formed a predominantly left-lateralized cortical pattern. In MCAS-R, lower DC involved right putaminal/insular, temporal, and parietal–sensorimotor regions, forming a predominantly right-lateralized pattern. Together, these findings suggest distributed alterations in functional centrality across temporal, frontal, parietal, sensorimotor, and subcortical regions rather than changes confined to a single anatomical area. Previous resting-state studies in ischemic stroke have similarly demonstrated alterations in DC across multiple brain regions (Min et al., 2023; Ding et al., 2024). Broader functional-connectivity studies have further demonstrated distributed network disruption and remote connectivity alterations after focal stroke (Wang et al., 2022; Jia et al., 2024; Katsurayama et al., 2024; Sagues et al., 2025). The present findings are consistent with a similar distributed-network perspective in patients with severe unilateral MCA stenosis accompanied by watershed infarction.

Higher DC showed different anatomical distributions in the two patient groups. In MCAS-R, increased DC was observed in a large left cerebellar cluster, whereas MCAS-L showed increased DC in right medial/subcortical and posterior-midline regions. These increases may reflect redistribution of functional centrality after ischemic injury; however, the present cross-sectional data cannot establish whether they represent compensatory neuronal reorganization.

Descriptive mapping of cluster peaks onto the Yeo seven-network parcellation provided additional functional-network context. Selected cortical peaks were affiliated with somatomotor, dorsal-attention, or ventral-attention networks, whereas cerebellar, subcortical, and several boundary peaks were not assigned to the cortical Yeo atlas. Because DC measures overall voxelwise connectivity strength rather than specific connections, these atlas affiliations should be interpreted descriptively and do not establish altered within-network or between-network connectivity.

### 4.3 Cognitive associations

The cognitive findings were among the clearest clinical features of the present study. Post-stroke cognitive impairment is increasingly understood in terms of disruption across distributed neural systems rather than damage to a single “cognitive center” (El Husseini et al., 2023; Li et al., 2026). Recent connectivity studies likewise link post-stroke cognitive deficits to remote or network-level functional alterations (Jia et al., 2024; Zhang et al., 2025b). Clinically relevant cognitive deficits may occur even when conventional neurological impairment is mild (Gallucci et al., 2024). In the present cohort, both patient groups had lower MMSE scores than HCs, whereas MoCA scores were lowest in MCAS-L, intermediate in MCAS-R, and highest in HCs. Together, these findings indicate measurable differences in global cognitive performance despite relatively mild neurological deficits.

Exploratory clusterwise associations were observed only in MCAS-R at the nominal significance level. Higher MMSE scores were associated with relatively higher DC in the right superior temporal-pole and right parietal–sensorimotor/supramarginal clusters, both of which showed lower DC relative to HCs. Conversely, MMSE scores were inversely associated with the higher-DC left cerebellar cluster. The differing directions of these nominal associations are compatible with a region-dependent relationship between BOLD-based functional centrality and cognitive performance, rather than a simple uniform “higher-is-better” pattern.

No nominal MoCA–DC associations were identified in either patient subgroup, and no nominal MMSE–DC associations were observed in MCAS-L. This does not necessarily conflict with the observed group differences in cognitive performance, because between-group comparisons and within-group correlations address different aspects of the data. Group comparisons characterize differences in cognitive performance between populations, whereas correlation analyses assess whether interindividual variation in cognitive scores covaries with DC within specific clusters. The nominal MMSE–DC associations observed in MCAS-R therefore provide exploratory evidence of region-specific covariation, but should not be regarded as confirmatory evidence of a cognitive mechanism.

The presence of nominal MMSE–DC associations in MCAS-R but not MCAS-L should not be interpreted as evidence of a general right-hemisphere predominance in cognitive–DC relationships. Rather, it may reflect subgroup-specific relationships between the spatial distribution of DC alterations and interindividual cognitive variation. Recent rs-fMRI work in acute ischemic stroke has also reported lesion-side-dependent differences in connectivity patterns (Katsurayama et al., 2024), underscoring that hemispheric effects may be complex and context dependent. Further studies combining domain-specific neuropsychological measures with longitudinal functional imaging will be needed to clarify whether these associations differ according to lesion side or cognitive domain.

Because the present study was cross-sectional and pre-stroke cognitive measurements were unavailable, the direction of these associations cannot be established. Pre-existing differences in cognitive reserve or baseline cognitive function may also have contributed. These associations should therefore be considered hypothesis-generating rather than causal.

### 4.4 Emotional symptoms and DC associations

Unlike cognitive performance, anxiety and depressive symptom scores did not differ significantly among the three groups. A single nominal association was observed in MCAS-R between HAMD scores and DC in a right middle temporal cluster. Previous rs-fMRI work has demonstrated distributed network alterations in post-stroke depression (Fan et al., 2023), while other studies have identified abnormalities involving spontaneous activity and reward/default-mode network interactions (Oestreich et al., 2022; Wu et al., 2023). However, given the absence of a group-level HAMD difference and the exploratory nature of the present correlation analysis, this finding should be regarded as hypothesis-generating and does not establish a region-specific depressive mechanism.

### 4.5 Clinical implications

The coexistence of relatively mild neurological deficits, measurable differences in cognitive performance, and distributed DC alterations may be clinically relevant. Structural imaging delineates ischemic lesions, whereas conventional neurological scales primarily characterize overt neurological impairment; voxelwise DC provides complementary information on the functional centrality of individual regions within the whole-brain network. In the present study, selected DC alterations showed exploratory nominal associations with MMSE performance in MCAS-R, suggesting that variation in functional centrality may warrant further investigation in relation to cognitive performance. These findings motivate further evaluation of DC as a candidate imaging feature for characterizing functional-centrality alterations associated with cognitive differences in this population. Longitudinal studies integrating DC with structural and vascular imaging and more detailed neuropsychological assessment are needed to determine its clinical relevance.

### 4.6 Limitations

Several limitations should be considered. First, the single-center, cross-sectional design and relatively modest patient-group sample sizes may limit generalizability and do not permit assessment of longitudinal changes in DC or cognition. Pre-stroke cognitive measurements were unavailable, which further limits interpretation of the direction of the observed clinical associations. Second, quantitative perfusion, cerebrovascular reactivity, and hemodynamic-delay measurements were unavailable. Therefore, neural and vascular contributions to the resting-state BOLD-based DC findings could not be fully separated (Braban et al., 2023; Wang et al., 2024). Third, DWI was available for confirming the presence and anatomical pattern of acute watershed infarction, but quantitative infarct-volume measurements and standardized individual lesion masks were not generated as part of the original study protocol. Consequently, infarct volume could not be incorporated as an imaging covariate, and direct voxelwise overlap between individual infarcts and significant DC clusters could not be quantified. We therefore cannot completely exclude an influence of local lesion burden on DC abnormalities located within or adjacent to watershed territories. Fourth, the clusterwise clinical correlations were exploratory follow-up analyses and were not adjusted for multiple comparisons. The reported *P* values therefore represent nominal significance levels. Accordingly, these associations should be interpreted cautiously and require independent confirmation in larger cohorts. Finally, MMSE and MoCA primarily assess global cognitive performance and do not provide a comprehensive domain-specific neuropsychological profile, whereas HAMA and HAMD quantify emotional symptoms rather than establish psychiatric diagnoses. Longitudinal multicenter studies incorporating quantitative vascular assessment, lesion-volume analysis, and domain-specific neuropsychological testing are needed.

## 5 Conclusion

Severe unilateral M1 MCA stenosis with ipsilateral watershed infarction was associated with distributed and lateralized alterations in voxelwise DC and reduced cognitive performance. Selected DC alterations showed exploratory nominal associations with MMSE performance in MCAS-R, whereas nominal cognitive–DC associations were not observed in MCAS-L. These findings may contribute to understanding functional-centrality alterations accompanying cognitive differences in this population. Longitudinal, larger-sample multimodal studies incorporating quantitative vascular and lesion assessment and domain-specific neuropsychological evaluation are needed to determine the stability and clinical relevance of these DC patterns.

## Data Availability

The datasets contain potentially sensitive clinical and neuroimaging information. De-identified data may be available from the corresponding author on reasonable request, subject to institutional and ethical approval and applicable data-protection requirements. Requests to access the datasets should be directed to  Jian Zhang, zhangjian@sr.gxmu.edu.cn.

## Footnotes

1 http://www.mathworks.com (Accessed April 2026).

2 http://www.fil.ion.ucl.ac.uk/spm (Accessed April 2026).
