# Supplementary material for: Voxelwise degree centrality alterations and cognitive performance in severe unilateral M1-segment middle cerebral artery stenosis with ipsilateral watershed infarction
Source: Front Aging Neurosci. 2026 Oct 7;18:1969092. doi: 10.3389/fnagi.2026.1969092 (PMC13683771; doi:10.3389/fnagi.2026.1969092)
Supplement: Supplementary file 1 [file Data_Sheet_1.docx]

Supplementary Material

CONTENTS

Supplementary Table S1. Extended anatomical composition of final GRF-corrected clusters in the education-adjusted primary DC analysis (r > 0.25)

Supplementary Table S2. Peak-coordinate functional-network affiliations of significant DC clusters according to the Yeo 2011 seven-network parcellation

Supplementary Table S3. Sensitivity analysis of degree-centrality findings across alternative correlation thresholds

Supplementary Table S1. Extended anatomical composition of final GRF-corrected clusters in the education-adjusted primary DC analysis (r > 0.25)

| **Contrast** | **Cluster** | **Direction** | **Total voxels** | **Peak AAL region(s)** | **Peak MNI (x, y, z)** | **Peak Z** | | **AAL regions within cluster, n (%)** |
| --- | --- | --- | --- | --- | --- | --- | --- | --- |
| MCAS-L vs HC | 1 | Increased | 94 | Unlabeled at peak | (15, 33, 9) | 5.0290 | Caudate_R, 12 (12.8%); Caudate_L, 3 (3.2%); Olfactory_L, 2 (2.1%); Olfactory_R, 1 (1.1%) | |
| MCAS-L vs HC | 2 | Increased | 54 | Unlabeled at peak | (9, -36, 6) | 4.9830 | Precuneus_R, 11 (20.4%); Cingulum_Post_R, 6 (11.1%); Hippocampus_R, 5 (9.3%); Thalamus_R, 2 (3.7%); Calcarine_R, 2 (3.7%); Lingual_R, 2 (3.7%); ParaHippocampal_R, 1 (1.9%) | |
| MCAS-L vs HC | 3 | Decreased | 67 | Rolandic_Oper_L | (-42, -21, 15) | −4.6469 | Temporal_Sup_L, 38 (56.7%); Rolandic_Oper_L, 15 (22.4%); Temporal_Mid_L, 12 (17.9%); SupraMarginal_L, 2 (3.0%) | |
| MCAS-L vs HC | 4 | Decreased | 177 | Temporal_Mid_L | (-48, -69, 6) | −5.2476 | Temporal_Mid_L, 130 (73.4%); Occipital_Inf_L, 20 (11.3%); Occipital_Mid_L, 15 (8.5%); Temporal_Sup_L, 9 (5.1%); Angular_L, 1 (0.6%); SupraMarginal_L, 1 (0.6%) | |
| MCAS-L vs HC | 5 | Decreased | 424 | Postcentral_L | (-48, -18, 39) | −5.5420 | Postcentral_L, 219 (51.7%); Parietal_Inf_L, 116 (27.4%); SupraMarginal_L, 29 (6.8%); Precentral_L, 16 (3.8%); Rolandic_Oper_L, 9 (2.1%); Angular_L, 6 (1.4%); Temporal_Sup_L, 5 (1.2%); Occipital_Mid_L, 3 (0.7%); Parietal_Sup_L, 2 (0.5%) | |
| MCAS-R vs HC | 1 | Increased | 241 | Cerebelum_8_L | (-6, -72, -42) | 5.1135 | Vermis_8, 37 (15.4%); Cerebelum_Crus1_L, 31 (12.9%); Vermis_6, 29 (12.0%); Cerebelum_8_L, 26 (10.8%); Vermis_7, 21 (8.7%); Cerebelum_Crus2_L, 13 (5.4%); Cerebelum_7b_L, 11 (4.6%); Cerebelum_8_R, 10 (4.1%); Cerebelum_6_L, 8 (3.3%); Cerebelum_6_R, 4 (1.7%); Vermis_10, 3 (1.2%); Vermis_9, 2 (0.8%); Cerebelum_7b_R, 2 (0.8%); Cerebelum_Crus1_R, 1 (0.4%); Vermis_4_5, 1 (0.4%); Cerebelum_4_5_L, 1 (0.4%) | |
| MCAS-R vs HC | 2 | Decreased | 114 | Unlabeled at peak | (33, -6, -12) | −5.6122 | Insula_R, 18 (15.8%); Putamen_R, 18 (15.8%); Amygdala_R, 11 (9.6%); Rolandic_Oper_R, 5 (4.4%); Temporal_Pole_Sup_R, 3 (2.6%); Hippocampus_R, 2 (1.8%); ParaHippocampal_R, 1 (0.9%) | |
| MCAS-R vs HC | 3 | Decreased | 109 | Temporal_Pole_Sup_R | (60, 3, -6) | −5.2127 | Temporal_Pole_Sup_R, 59 (54.1%); Temporal_Sup_R, 37 (33.9%); Rolandic_Oper_R, 9 (8.3%); Temporal_Pole_Mid_R, 2 (1.8%); Frontal_Inf_Orb_R, 1 (0.9%) | |
| MCAS-R vs HC | 4 | Decreased | 160 | Temporal_Mid_R | (54, -54, 9) | −5.2924 | Temporal_Mid_R, 113 (70.6%); Occipital_Mid_R, 26 (16.2%); Angular_R, 7 (4.4%); Temporal_Sup_R, 4 (2.5%); SupraMarginal_R, 4 (2.5%) | |
| MCAS-R vs HC | 5 | Decreased | 180 | Temporal_Sup_R | (54, -33, 12) | −5.5670 | Temporal_Sup_R, 118 (65.6%); Heschl_R, 13 (7.2%); Insula_R, 13 (7.2%); SupraMarginal_R, 9 (5.0%); Rolandic_Oper_R, 9 (5.0%); Hippocampus_R, 1 (0.6%) | |
| MCAS-R vs HC | 6 | Decreased | 400 | SupraMarginal_R | (48, -27, 36) | −6.0021 | Postcentral_R, 98 (24.5%); Parietal_Inf_R, 86 (21.5%); Precentral_R, 57 (14.2%); SupraMarginal_R, 57 (14.2%); Angular_R, 21 (5.2%); Parietal_Sup_R, 4 (1.0%); Rolandic_Oper_R, 1 (0.2%) | |

Note: Only AAL labels explicitly reported in the final corrected cluster reports are shown. Counts and percentages represent the voxel overlap between each GRF-corrected cluster and individual AAL parcels, with percentages calculated relative to the total corrected cluster extent; percentages may not sum to 100% because some cluster voxels were not assigned to an AAL parcel. Peak AAL region(s) correspond to the AAL label returned at the peak MNI coordinate; “Unlabeled at peak” indicates that no AAL parcel was assigned at that peak coordinate, although AAL overlaps within the cluster are still reported for anatomical context. The primary analysis used weighted DC at r > 0.25 and the education-adjusted voxelwise model. GRF correction was applied at a two-tailed voxel-level threshold of P < 0.001 and cluster-level threshold of P < 0.05 within the brain mask; the GRF-derived minimum cluster extents were 50 voxels for MCAS-L versus HC and 53 voxels for MCAS-R versus HC. AAL, Automated Anatomical Labeling; DC, degree centrality; GRF, Gaussian random field; HC, healthy control; MCAS-L, left-sided severe M1-segment middle cerebral artery stenosis group; MCAS-R, right-sided severe M1-segment middle cerebral artery stenosis group; MNI, Montreal Neurological Institute.

Supplementary Table S2. Peak-coordinate functional-network affiliations of significant DC clusters according to the Yeo 2011 seven-network parcellation

| **Contrast** | **Cluster** | **Direction** | **Voxels** | **Peak MNI** | **Peak AAL label** | **Yeo-7 network at peak** |
| --- | --- | --- | --- | --- | --- | --- |
| MCAS-L vs HC | 1 | Increased | 94 | (15, 33, 9) | Unlabeled at peak | Not assigned |
| MCAS-L vs HC | 2 | Increased | 54 | (9, −36, 6) | Unlabeled at peak | Not assigned |
| MCAS-L vs HC | 3 | Decreased | 67 | (−42, −21, 15) | Rolandic_Oper_L | Somatomotor |
| MCAS-L vs HC | 4 | Decreased | 177 | (−48, −69, 6) | Temporal_Mid_L | Dorsal Attention |
| MCAS-L vs HC | 5 | Decreased | 424 | (−48, −18, 39) | Postcentral_L | Somatomotor |
| MCAS-R vs HC | 1 | Increased | 241 | (−6, −72, −42) | Cerebelum_8_L | Not assigned |
| MCAS-R vs HC | 2 | Decreased | 114 | (33, −6, −12) | Unlabeled at peak | Not assigned |
| MCAS-R vs HC | 3 | Decreased | 109 | (60, 3, −6) | Temporal_Pole_Sup_R | Somatomotor |
| MCAS-R vs HC | 4 | Decreased | 160 | (54, −54, 9) | Temporal_Mid_R | Ventral Attention |
| MCAS-R vs HC | 5 | Decreased | 180 | (54, −33, 12) | Temporal_Sup_R | Somatomotor |
| MCAS-R vs HC | 6 | Decreased | 400 | (48, −27, 36) | SupraMarginal_R | Not assigned |

Note: Functional-network affiliation was determined in DPABI_VIEW using the peak MNI coordinate of each GRF-corrected cluster and the Yeo 2011 seven-network cortical parcellation. Peak coordinates falling outside the cortical Yeo-7 parcellation were classified as “Not assigned.” These labels provide complementary functional-network context and do not represent direct evidence of altered within-network or between-network connectivity.

Supplementary Table S3. Sensitivity analysis of degree-centrality findings across alternative correlation thresholds

| **Threshold** | **Contrast** | **Cluster** | **Direction** | **Cluster extent, voxels** | **Peak MNI (x, y, z)** | **Peak Z** | **Peak AAL label / major AAL overlap*** |
| --- | --- | --- | --- | --- | --- | --- | --- |
| r > 0.20 | MCAS-L vs HC | 1 | Increased | 55 | −33, −45, −39 | 4.7694 | Cerebelum_8_L† |
| r > 0.20 | MCAS-L vs HC | 2 | Decreased | 197 | −48, −69, 6 | −5.3370 | Temporal_Mid_L |
| r > 0.20 | MCAS-L vs HC | 3 | Increased | 109 | 18, 33, 9 | 5.2551 | Caudate_R† |
| r > 0.20 | MCAS-L vs HC | 4 | Increased | 65 | 9, −36, 6 | 5.1737 | Precuneus_R† |
| r > 0.20 | MCAS-L vs HC | 5 | Decreased | 647 | −48, −18, 39 | −5.8187 | Postcentral_L |
| r > 0.20 | MCAS-L vs HC | 6 | Increased | 55 | 21, −18, 18 | 4.9631 | Thalamus_R† |
| r > 0.20 | MCAS-R vs HC | 1 | Increased | 128 | 27, −39, −39 | 5.0312 | Cerebelum_8_R† |
| r > 0.20 | MCAS-R vs HC | 2 | Increased | 270 | −3, −72, −45 | 5.2295 | Vermis_8† |
| r > 0.20 | MCAS-R vs HC | 3 | Decreased | 741 | 33, −6, −12 | −5.9504 | Temporal_Sup_R† |
| r > 0.20 | MCAS-R vs HC | 4 | Increased | 126 | −21, 42, 0 | 4.7328 | Frontal_Mid_Orb_L / Caudate_L†‡ |
| r > 0.20 | MCAS-R vs HC | 5 | Decreased | 457 | 48, −27, 36 | −6.0980 | SupraMarginal_R |
| r > 0.25 | MCAS-L vs HC | 1 | Increased | 94 | 15, 33, 9 | 5.029 | Caudate_R† |
| r > 0.25 | MCAS-L vs HC | 2 | Increased | 54 | 9, −36, 6 | 4.983 | Precuneus_R† |
| r > 0.25 | MCAS-L vs HC | 3 | Decreased | 67 | −42, −21, 15 | −4.6469 | Rolandic_Oper_L |
| r > 0.25 | MCAS-L vs HC | 4 | Decreased | 177 | −48, −69, 6 | −5.2476 | Temporal_Mid_L |
| r > 0.25 | MCAS-L vs HC | 5 | Decreased | 424 | −48, −18, 39 | −5.5420 | Postcentral_L |
| r > 0.25 | MCAS-R vs HC | 1 | Increased | 241 | −6, −72, −42 | 5.1135 | Cerebelum_8_L |
| r > 0.25 | MCAS-R vs HC | 2 | Decreased | 114 | 33, −6, −12 | −5.6122 | Insula_R / Putamen_R†‡ |
| r > 0.25 | MCAS-R vs HC | 3 | Decreased | 109 | 60, 3, −6 | −5.2127 | Temporal_Pole_Sup_R |
| r > 0.25 | MCAS-R vs HC | 4 | Decreased | 160 | 54, −54, 9 | −5.2924 | Temporal_Mid_R |
| r > 0.25 | MCAS-R vs HC | 5 | Decreased | 180 | 54, −33, 12 | −5.5670 | Temporal_Sup_R |
| r > 0.25 | MCAS-R vs HC | 6 | Decreased | 400 | 48, −27, 36 | −6.0021 | SupraMarginal_R |
| r > 0.30 | MCAS-L vs HC | 1 | Decreased | 161 | −48, −69, 6 | −5.0626 | Temporal_Mid_L |
| r > 0.30 | MCAS-L vs HC | 2 | Decreased | 50 | −42, −21, 15 | −4.5475 | Rolandic_Oper_L |
| r > 0.30 | MCAS-L vs HC | 3 | Increased | 53 | 15, 33, 9 | 4.8221 | Caudate_R† |
| r > 0.30 | MCAS-L vs HC | 4 | Decreased | 289 | −45, −21, 33 | −5.2522 | SupraMarginal_L |
| r > 0.30 | MCAS-R vs HC | 1 | Increased | 179 | −6, −72, −42 | 4.9801 | Cerebelum_8_L |
| r > 0.30 | MCAS-R vs HC | 2 | Decreased | 87 | 33, −6, −12 | −5.1474 | Putamen_R† |
| r > 0.30 | MCAS-R vs HC | 3 | Decreased | 77 | 60, 0, −6 | −5.0764 | Temporal_Sup_R |
| r > 0.30 | MCAS-R vs HC | 4 | Decreased | 130 | 54, −54, 9 | −5.0236 | Temporal_Mid_R |
| r > 0.30 | MCAS-R vs HC | 5 | Decreased | 113 | 54, −33, 12 | −5.2729 | Temporal_Sup_R |
| r > 0.30 | MCAS-R vs HC | 6 | Decreased | 344 | 48, −27, 36 | −5.6525 | SupraMarginal_R |

Note: The r > 0.25 threshold was used for the primary analysis. Sensitivity analyses were additionally performed using r > 0.20 and r > 0.30, while all other preprocessing procedures, covariates, analysis masks, group-comparison procedures, and GRF correction thresholds were kept identical to those of the primary analysis. Anatomical labels were derived directly from the DPABI GRF-corrected cluster reports. When an AAL label was available at the peak MNI coordinate, that label was reported. When no AAL parcel was assigned to the peak coordinate, the AAL parcel with the largest voxel overlap within the corresponding cluster was reported. AAL, Automated Anatomical Labeling; DC, degree centrality; GRF, Gaussian random field; HC, healthy controls; MCAS-L, left-sided severe M1-segment middle cerebral artery stenosis group; MCAS-R, right-sided severe M1-segment middle cerebral artery stenosis group; MNI, Montreal Neurological Institute.

† The peak MNI coordinate was not assigned to an AAL parcel; therefore, the AAL parcel with the largest voxel overlap within the cluster is shown.

‡ Two AAL parcels had the same largest voxel overlap and are both reported.
